# Corticosterone, inflammation, immune status and telomere length in frigatebird nestlings facing a severe herpesvirus infection

**DOI:** 10.1093/conphys/cow073

**Published:** 2017-01-04

**Authors:** Manrico Sebastiano, Marcel Eens, Frederic Angelier, Kévin Pineau, Olivier Chastel, David Costantini

**Affiliations:** 1 Behavioural Ecology and Ecophysiology group, Department of Biology, University of Antwerp, Universiteitsplein 1, 2610 Wilrijk, Belgium; 2 Centre d'Etudes Biologiques de Chizé (CEBC), UMR7372 – CNRS/Université La Rochelle, F-79360, France; 3 Groupe d'Etude et de Protection des Oiseaux en Guyane (GEPOG), 15 Avenue Pasteur 97300 Cayenne, France; 4 Leibniz Institute for Zoo and Wildlife Research, Alfred-Kowalke-Straße 17, 10315 Berlin, Germany; 5 UMR 7221, Muséum National d'Histoire Naturelle, 7 rue Cuvier, 75231 Paris cedex 05, France

**Keywords:** Corticosterone, herpesvirus, immunocompetance, telomere length

## Abstract

Herpesvirus outbreaks are common in natural animal populations, but little is known about factors that favour the infection and its consequences for the organism. In this study, we examined the pathophysiological consequences of a disease probably attributable to herpesvirus infection for several markers of immune function, corticosterone, telomere length and inflammation. In addition, we assessed whether any markers used in this study might be associated with the occurrence of visible clinical signs of the disease and its impact on short-term survival perspectives. To address our questions, in spring 2015, we collected blood samples from nestlings of the magnificent frigatebird (*Fregata magnificens*) that were free of any clinical signs or showed visible signs of the disease. We found that the plasma concentration of haptoglobin was strongly associated with the infection status and could predict probabilities of survival. We also found that nestlings with clinical signs had lower baseline corticosterone concentrations and similar telomere length compared with healthy nestlings, whereas we did not find any association of the infection status with innate immune defenses or with nitric oxide concentration. Overall, our results suggest that the plasma concentration of haptoglobin might be a valuable tool to assess survival probabilities of frigatebird nestlings facing a herpesvirus outbreak.

## Abstract (precis)

In this study, we examined the pathophysiological consequences likely to be attributable to a herpesvirus infection for immune function, corticosterone, telomere length and inflammation. We found that the plasma concentration of haptoglobin was strongly associated with the observable clinical manifestation of disease and could predict probabilities of survival.

## Introduction

Although organisms have evolved a range of specific and non-specific mechanisms of protection against pathogens, there are circumstances (e.g. food shortage, high stress level) that make the organism unable to control the activity of a given pathogen (Klimpel, 1996; van Boven and Weissing, 2004). Understanding the way in which free-living animals modulate their response to cope with pathogens is therefore of great ecological relevance and might even provide conservation practitioners with tools to predict the impact of a pathogen on fitness traits. For example, a previous study found that the concentration of haptoglobin (an inflammation-inducible protein) was lower in Japanese quail (*Coturnix japonica*) that died from *Aspergillus fumigatus* infection in comparison with individuals that have survived (Goetting *et al*., 2013) and showed how haptoglobin may be used to describe survival probabilities (Goetting *et al*., 2013). Moreover, as the levels of natural antibodies and complement in small blood samples provide information about immunocompetence (Matson *et al*., 2005), these markers have been used to quantify the innate immunity of vertebrates in stressful conditions (Hegemann *et al*., 2013; Vermeulen *et al*., 2016).

Herpesviruses are one of the most common infectious pathogens in humans and in both wild and domestic animals. However, little is known about the causes and pathophysiological consequences of herpesvirus infection in wild animals (Goldberg *et al*., 1990). Decreased immunocompetence appears to favour outbreaks of herpesvirus (Goldberg *et al*., 1990). The causes of a reduction in immunocompetence might lie with sources of environmental stress; for example, chronic secretion of glucocorticoid hormones in response to a stressful situation can reduce the individual's capacity to mount an immune response (Sapolsky *et al*., 2000). Specifically, environmental stressful stimuli activate the hypothalamic–pituitary–adrenal axis, which leads to the release of corticotrophin-releasing factors in the brain and glucocorticoids in the periphery (Romero, 2004). The action of glucocorticoids on the immune system is dependent on the duration of the stress exposure. For example, it was found that a short-term exposure to a stressful experience enhanced the immune response, whereas a prolonged exposure reduced immunocompetence (Dhabhar and McEwen, 1997; Dhabhar, 2000). In birds, elevated concentrations of corticosterone (CORT; the main avian glucocorticoid) have detrimental effects on nestling growth, behaviour and immunity (Kitaysky *et al*., 2003; Rubolini *et al*., 2005) and can even increase the generation of reactive oxygen species (Costantini *et al*., 2011). Both chronic stressful conditions and generation of reactive oxygen species via CORT negatively affect the dynamics of the telomeres (von Zglinicki, 2002; Kotrschal *et al*., 2007; Haussmann *et al*., 2012; Quirici *et al*., 2016), long repetitive non-coding sequences of DNA located at the ends of chromosomes, which can play a major role in ageing processes (Blackburn, 1991). Telomeres, considered as valuable indicators of cell health (Counter, 1996), might also reflect the individual's ability to cope with stressful conditions (Kotrschal *et al*., 2007) and predict lifespan, reproductive outcome or survival perspective in birds (Haussmann *et al*., 2005; Heidinger *et al*., 2012; Angelier *et al*., 2013).

In individuals exposed to a herpesvirus outbreak, chronic activation of the hypothalamic–pituitary–adrenal axis might favour viral activity (Padgett and Glaser, 2003); for example, through an impairment of the immune response (Casto *et al*., 2001; Stier *et al*., 2009; Vallverdú-Coll *et al*., 2016). The assessment of stress-induced CORT is therefore of crucial importance for the magnificent frigatebird (*Fregata magnificens*; hereafter called frigatebird) population used in this study, which is probably undergoing a strong exposure to environmental stressors (Martinet and Blanchard, 2009; Sebastiano *et al*., 2016). For instance, a study on experimentally infected mice found that herpes simplex virus-associated morbidity and mortality during development are increased by stress-induced CORT (Elftman *et al*., 2010). However, there is a lack of studies addressing the physiological consequences of a herpesvirus infection on individuals’ stress levels and its deleterious effect on immunity and telomeres in wild animals. Furthermore, there is an urgent need to identify markers associated with herpesvirus infections that can be used for the assessment of health status in order to strengthen conservation strategies. For instance, as infectious diseases may induce inflammation (Eckersall *et al*., 2001; Gånheim *et al*., 2007; Grau-Roma *et al*., 2009; White *et al*., 2012), inflammatory markers might be a tool to diagnose and monitor animal diseases (Jain *et al*., 2011; Goetting *et al*., 2013).

In this study, we measured the basal plasma concentration of CORT, telomere length and an array of immune and inflammatory markers in frigatebird nestlings likely to be affected by a severe herpesvirus infection (Sebastiano *et al*., 2016). The aims of this study were as follows: (i) to investigate whether the chances of showing visible clinical signs of the disease are higher in immunosuppressed individuals; (ii) to assess whether individuals with visible signs have higher physiological stress and inflammation than those individuals without any clinical signs; (iii) to assess whether short-term survival probabilities are related to physiological markers; (iv) to assess the sensitivity and specificity of immunological and inflammatory markers for diagnosis of virus infections; and (v) to assess whether telomere length might be associated with the infection status.

## Materials and methods

### Study site and sampling

**Figure 1:**
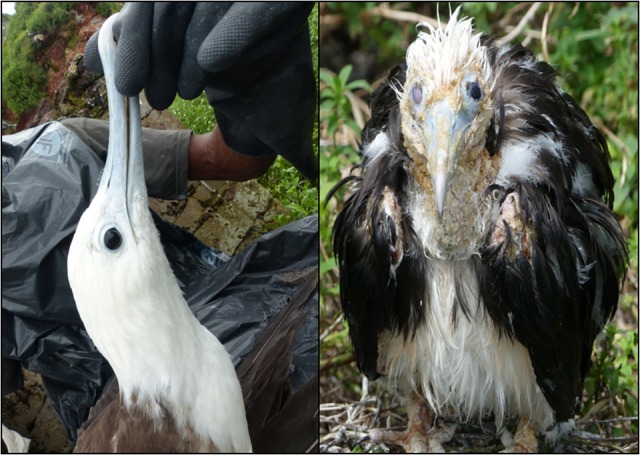
A healthy nestling, on the left, does not show visible clinical signs of the disease (hyperkeratosis, body and head crusts) in comparison to a sick nestling, on the right.

Grand Connétable island is a protected area located off the Northern Atlantic coast of South America (French Guiana, 4°49′30N, 51°56′00W). This island hosts a unique colony of frigatebirds that is one of the most important in South America and represents the only breeding site for frigatebirds in French Guiana (Dujardin and Tostain, 1990). A total of 44 nestlings, including 22 nestlings without clinical signs of herpesvirus infection and 22 nestlings with visible clinical signs of a herpesvirus infection as previously described (de Thoisy *et al*., 2009) of ~4 months old (termed ‘sick’; Fig. 1), were captured by hand during the breeding season of 2015, on 5–7 June, and recaptured on 19–21 June. As sick nestlings vary in the severity of visible clinical signs (probably because of a different stage of the infection), we chose sick nestlings with the same severity of visible clinical signs. In the second sampling period, of the nestlings that were previously blood sampled nine were found dead from the disease and four were not found. In addition, six new nestlings were sampled. Out of the 22 apparently healthy individuals sampled during the first period, six nestlings showed the occurrence of clinical signs in the second period.

Within 3 min after capture, 2 ml of blood was collected from the brachial vein using a heparinized syringe and a 25 gauge needle. After removing the needle from the syringe (in order to avoid haemolysis), blood was carefully transferred into a 2 ml tube and immediately kept cold (in a box with ice packs). Blood was then brought back to the field station within a few minutes of collection and was centrifuged within 1 h to separate plasma from red blood cells. After centrifugation, plasma and red blood cells were divided further into several tubes in order to avoid repeated thawing for laboratory analyses. All the tubes were kept on dry ice until the end of the fieldwork and, when back at the laboratory, were kept in a −80°C freezer until laboratory analyses.

### Haemolysis–haemagglutination assay

To characterize constitutive innate humoral immunity, we used the haemolysis–haemagglutination assay as described in a previous protocol (Matson *et al*., 2005). In this assay, serially diluted plasma of frigatebird nestlings was evaluated for its ability to lyse and agglutinate exogenous red blood cells from rabbits (Matson *et al*., 2005). Lysis reflects the interaction of complement and natural antibodies, whereas agglutination results from natural antibodies only. From the digitalized images, lysis and agglutination were scored twice for each sample and recorded as the negative log_2_ of the last plasma dilution at which agglutination or lysis occurred. Titres that showed intermediate agglutination or lysis values were assigned half score. All plasma samples were scored twice; the coefficient of variation was 4.9% for agglutination and 4.3% for lysis, respectively.

### Haptoglobin and nitric oxide assays

The concentration of plasma haptoglobin (an inflammation-inducible protein; expressed as milligrams per millilitre) was quantified using the manufacturer's instructions of a commercially available assay (PHASE Haptoglobin assay; Tridelta Development Ltd), which quantifies the haem-binding capacity of haptoglobin colorimetrically. In each plate, a standard curve and an internal standard were run in duplicate.

The plasma concentration of nitric oxide (in micromoles per litre), used in immunoecological research for assessment of the magnitude of the inflammatory response and potential immunopathological damage (Sild and Hõrak, 2009), was estimated from the concentration of nitrate and nitrite, the stable end products of nitric oxide oxidation. The principle of the assay is the reduction of nitrate to nitrite by copper-coated cadmium granules, followed by colour development with Griess reagent (Sild and Hõrak, 2009).

### Telomere assay

Determination of telomere length was performed at the Centre d'Etudes Biologiques de Chizé (CEBC) by Southern blot using the TeloTAGGG Telomere Length Assay (Roche, Mannheim, Germany) as previously described (Blévin *et al*., 2016). After digestion with proteinase K and DNA extraction from red blood cells using the DNeasy blood and tissue kit (Qiagen), DNA quality was carefully checked before telomere analyses (Nussey *et al*., 2014). Following extraction, DNA yield and purity were checked using a spectrophotometer (Nanodrop ND-1000; Thermo Scientific, USA). All sample yields were >20 ng µl^−1^. Moreover, ranges for absorption of all samples were within an acceptable range (between 1.8 and 2.0 for 260 nm/280 nm ratio and between 1.9 and 2.2 for 260 nm/230 nm ratio). DNA was digested with the restriction enzymes HinfI and RsaI for 16 h at 37°C, and DNA samples were subsequently separated using a pulse-field gel electrophoresis (Bio-Rad) on a 0.8% agarose gel. Samples were randomly assigned to a gel and run in four gels, and inter-gel variations were measured. The gels were run at 3.0 V/cm, with an initial switch time of 0.5 s to a final switch time of 7 s for 14 h. The gels were then depurated and denaturized in an alkaline solution. The gels were then neutralized, and DNA was transferred onto a nitrocellulose membrane by Southern blot (Hybond N+; Amersham Life Science, Amersham, UK), which was incubated at 120°C for 20 min in order to fix the DNA. The DNA was then hybridized with a digoxigenin-labelled probe specific for telomeric sequences and incubated with antidigoxigenin-specific antibody prior to visualization with a Chemidoc (Bio-Rad). Estimation of telomere length was then performed using ImageJ to extract telomere smear densities. The lane-specific background was subtracted from each density value, and the telomere length (mean value) was then calculated using a window of 7–30 kb, and the inter-gel coefficient of variation was 2.0%.

### Sex determination

At the CEBC, DNA samples were used to determine the sex of individuals by polymerase chain reaction amplification as previously detailed, with minor modifications (Griffiths *et al*., 1998). Sex identification was carried out using the P8 (5′-CTCCCAAGGATGAGRAAYTG-3′) and P2 (5′-TCTGCATCGCTAAATCCTTT-3′) primers. An initial denaturing step at 94°C for 2 min was followed by 40 cycles of 94° for 30 s, 48°C for 30 s, and 72°C for 1 min. A final run of 94°C for 30 s, 50°C for 1 min, and 72°C for 5 min completed the programme.

### Corticosterone assay

Corticosterone concentrations were determined at the CEBC following a previous protocol (Lormée *et al*., 2003) for steroid hormones. Plasma CORT was measured in samples after ethyl ether extraction by radioimmunoassays using a commercial antiserum, raised in rabbits against corticosterone-3-(*O*-carboxy-methyl) oxime bovine serum albumin conjugate (Biogenesis, UK). Corticosterone concentrations showed no significant relationship with the hour of sampling (*r* = 0.18, *P* = 0.10, *n* = 81), and were all collected within 3 min after capture, thus they were considered to reflect the baseline levels. The intra-assay variation was 6.4% (*n* = 6 duplicates).

### Statistics

All statistical analyses were performed using R (version 3.1.2). Given that out of the 22 healthy-looking nestlings sampled during the first period, six nestlings showed visible clinical signs (hyperkeratosis, body and head crusts) during the second sampling period, our data set was divided into three different groups. The first group included nestlings without visible signs of the disease in both sampling periods (hereafter called ‘healthy’; *n* = 16). The second group included nestlings that manifested clinical signs only in the second sampling period (hereafter called ‘activated’; *n* = 6), and the third group contained nestlings that already showed clinical signs during the first sampling period (hereafter called ‘sick’; *n* = 22).

Haptoglobin, nitric oxide, CORT and telomere length were included as dependent variables in separate linear mixed models; group (healthy, activated and sick) and sampling period were included as fixed factors, and the factor ‘individual’ was included as a random effect because we had repeated measurements. Outcomes of all models were unchanged if the sampling time (calculated as minutes elapsed since midnight) was included. Thus, we present outcomes of models that do not take into account sampling time. In each model, we also included the interaction between the group and the sampling period. All individuals for which we had repeated measurements (nestlings captured in the first sampling period and recaptured in the second sampling period; *n* = 31) were included. This approach was chosen in order to account for differences among groups during the first and/or the second sampling period, as well as to determine the ‘change’ (a significant decrease or increase in the specific biomarker) of each group from the first to the second period.

Haemagglutination and haemolysis scores were included as dependent variables in two separate generalized linear mixed models with a binomial error distribution; in these models, we included the same fixed and random factors as for the linear mixed models. Generalized linear mixed models were used because the dependent variables were expressed as count data. Laboratory analyses were not successful for three individuals that were therefore excluded from the statistical analyses. All individuals for which we had repeated measurements (nestlings captured in the first sampling period and recaptured in the second sampling period; *n* = 28) were included.

We removed the interaction term from each full model when it was not significant in order to attain the best-fit model, which therefore included fixed factors only.

Sex differences in the specific biomarker were evaluated for both sampling periods separately, in order to include the individuals that were sampled only in one period. The model for data collected in the first sampling period also included the individuals that were found either dead because of the disease or that were not been found (*n* = 44; 22 healthy and 22 sick), whereas the second model included individuals sampled in the second period only (*n* = 37; 31 recaptured plus six new individuals). In this model, we also included the interaction between sex and group to test whether any differences between males and females depended on the health status. This model also enabled us to assess whether there were any differences among groups within each sampling period. In both the first and the second sampling period, two groups were present (‘with’ or ‘without’ clinical signs). In these models, the activated group was not present because individuals from this group did not show clinical signs at the first sampling period (and were therefore included in the ‘without clinical signs’ group), and in the second sampling period they showed clinical signs (and were therefore included in the ‘with clinical signs’ group). The significant differences that were found between individuals ‘with’ or ‘without’ clinical signs are reported in the Results section. Dependent variables were tested for normality with a Shapiro–Wilk test and were logarithmically transformed when necessary. Models were also tested for heteroscedasticity and normality of residuals. For each model, we removed non-significant interaction first, and then each non-significant factor in order to attain the best-fit model.

The probabilities of each marker to predict the occurrence of clinical signs and short-term survival were estimated using a generalized linear model with a logit link function and a binomial error variance. To predict the occurrence of clinical signs, we used the 22 healthy individuals in the first sampling period (six of which showed clinical signs during the second sampling period). The survival perspectives within our study period were estimated using two different models, as follows: the first model included 22 sick individuals sampled during the first period, and the second model included all the 44 individuals sampled during the first period. In survival models, individuals that have been found in the second period (*n* = 4) were not considered as dead in the model (thus, the comparison was made by using the nine nestlings that died from herpesvirus disease, eight of which were already showing clinical signs in the first sampling period). In these models, we have considered as ‘1’ those individuals that were alive in the second sampling period and ‘0’ those individuals that were found dead. An estimation of overdispersion has been performed to minimize the risk of type I error (Hilbe, 2011), and any data transformation to achieve normality is reported when needed.

Furthermore, haptoglobin and nitric oxide values were also used for diagnosis of herpesvirus. Haptoglobin was chosen because previous studies have shown that the concentration of haptoglobin changes significantly in response to an infection, injury or malignancy (Quaye, 2008) and can increase or decrease depending on the type of infection (Georgieva *et al*., 2010; Goetting *et al*., 2013). For those reasons, haptoglobin has recently been proposed as a predictor of both clinical signs and survival in diverse diseases in humans and birds (Goetting *et al*., 2013; Sun *et al*., 2016). We chose nitric oxide because its concentration is related to that of haptoglobin (Schaer *et al*., 2016) and because previous studies suggested the use of nitric oxide as a measure of innate immunity (Bourgeon *et al*., 2007) and a condition index during pathogenesis (Lillehoj and Li, 2004). The idea of a diagnostic test is to increase (or decrease) the suspicion that an individual has a particular disease (Parikh *et al*., 2008). The sensitivity reflects the ability of a test to classify an individual correctly as ‘diseased’, and in this study sensitivity was expressed as the percentage of nestlings correctly identified as showing the occurrence of clinical signs over time. The specificity reflects the ability of a test to classify an individual correctly as disease free, and in this study specificity was expressed as the percentage of nestlings correctly identified as not showing clinical signs over time, calculated on apparently healthy individuals. The use of these tests in animals affected by infectious diseases is increasing (Goetting *et al*., 2013).

## Results

### Haptoglobin and nitric oxide

**Figure 2:**
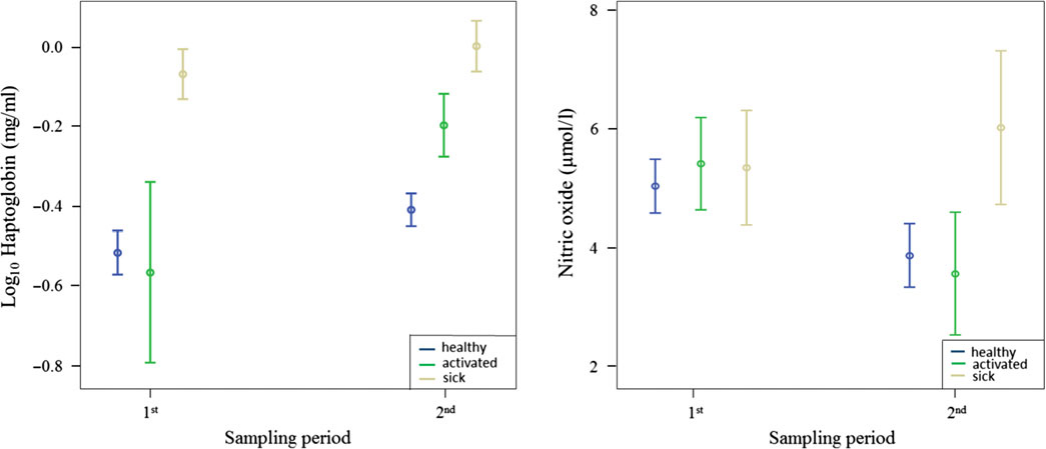
Mean values ± SEM of the haptoglobin and nitric oxide concentrations between the first and the second sampling period. Groups are shown in the following order: healthy, activated, and sick.

The model of haptoglobin concentration in plasma showed significant differences among groups and between sampling periods (Fig. 2 and Tables 1 and 2), but the interaction between group and sampling period was not significant (Fig. 2 and Tables 1 and 2). The haptoglobin concentration was higher in the sick group than in the healthy group (*P* < 0.01) and the activated group (*P* < 0.01), whereas there were no significant differences between the healthy group and the activated group. The haptoglobin content generally increased over time (*P* < 0.01). Males and females did not differ in haptoglobin concentration both in the first (*F* = 0.06, *P* = 0.81) and in the second sampling period (*F* = 0.01, *P* = 0.93). Individuals with clinical signs had significantly higher concentrations of haptoglobin than individuals without clinical signs both in the first (*t* = 6.24, *P* < 0.01) and in the second sampling period (*t* = 3.64, *P* < 0.01).

**Table 1:** Mean values ± SD of the inflammation markers, immune response, corticosterone and telomere length of each group in both sampling periods

Biomarker	First sampling period			Second sampling period		
	Healthy (mean ± SD)	Activated (mean ± SD)	Sick (mean ± SD)	Healthy (mean ± SD)	Activated (mean ± SD)	Sick (mean ± SD)
Telomere length (kb)	11.0 ± 0.5	11.1 ± 0.2	11.3 ± 0.4	10.9 ± 0.4	11.2 ± 0.3	11.4 ± 0.4
Haemagglutination	6.0 ± 1.6	6.5 ± 1.0	6.4 ± 1.5	5.7 ± 1.0	6.3 ± 0.4	6.4 ± 1.5
Haemolysis	4.6 ± 1.5	4.7 ± 0.9	5.0 ± 1.3	4.4 ± 0.9	5.1 ± 0.4	5.3 ± 1.3
Haptoglobin (mg/ml)	0.32 ± 0.10	0.41 ± 0.30	0.95 ± 0.55	0.40 ± 0.13	0.67 ± 0.28	1.09 ± 0.48
Nitric oxide (µmol/l)	5.0 ± 1.5	5.4 ± 1.8	5.3 ± 3.6	3.9 ± 1.7	3.6 ± 2.5	6.0 ± 4.8
Corticosterone (ng/ml)	27.3 ± 20.7	21.9 ± 26.3	16.9 ± 9.1	49.4 ± 33.6	22.0 ± 14.1	20.4 ± 18.9

**Table 2:** Full and final linear mixed models of inflammation markers, telomere length and corticosterone, and full and final generalized linear mixed models of the immune response markers

		Full model			Final model		
Variable	Effect	d.f.	*F*-value	*P*-value	d.f.	*F*-value	*P*-value
**Inflammation**							
Haptoglobin	Group	2,28	12.83	**<0.01**	2,28	12.83	**<0.01**
	Period	1,28	13.30	**<0.01**	1,30	8.04	**<0.01**
	Group*period	2,28	2.89	0.07			
Nitric oxide	Group	2,28	0.49	0.62	2,28	0.50	0.62
	Period	1,28	4.77	**0.04**	1,30	2.48	0.13
	Group*period	2,28	2.31	0.12			
**Immunity**							
Haemagglutination	Group	2,25	0.45	0.64	2,25	0.45	0.64
	Period	1,25	0.11	0.75	1,27	0.11	0.75
	Group*period	2,25	0.01	0.99			
Haemolysis	Group	2,25	0.01	0.99	2,25	0.01	0.99
	Period	1,25	<0.01	0.99	1,27	<0.01	0.99
	Group*period	2,25	0.05	0.95			
**Damage**							
Telomere length	Group	2,28	2.72	0.08	2,28	2.72	0.08
	Period	1,28	0.09	0.76	1,30	0.11	0.74
	Group*period	2,28	1.14	0.33			
**Hormone**							
Corticosterone	Group	2,28	3.36	**<0.05**	2,28	3.36	**<0.05**
	Period	1,28	0.69	0.41	1,30	0.44	0.51
	Group*period	2,28	0.59	0.56			

Significant *P*-values are shown in bold.

The nitric oxide concentration did not differ among groups, nor did it show any significant changes over time (Fig. 2 and Tables 1 and 2). Males and females did not differ in nitric oxide concentrations both in the first (*F* = 0.06, *P* = 0.80) and in the second sampling period (*F* = 0.54, *P* = 0.47).

Sensitivity and specificity were used to compare the diagnostic power of haptoglobin and nitric oxide, and results are shown in Table 3. The haptoglobin test had higher sensitivity and specificity than nitric oxide for herpesvirus infection. Taken together, haptoglobin and nitric oxide reached a sensitivity of 100%, while specificity dropped (Table 3).

**Table 3:** Diagnostic sensitivity and specificity of inflammatory markers for herpesvirus infection

Marker	Positive test value	Sensitivity (%)	Specificity (%)
Haptoglobin	>0.39 mg/ml	67	73
Nitric oxide	<0.05 µmol/l	67	45
Haptoglobin + nitric oxide	Haptoglobin >0.39 mg/ml or nitric oxide <0.05 µmol/l	100	45

### Haemolysis and haemagglutination tests

**Figure 3:**
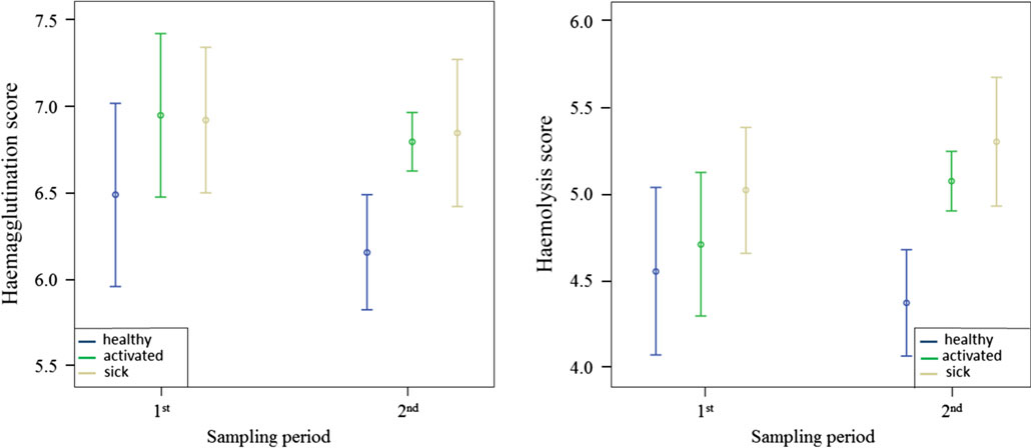
Mean values ± SEM of the haemagglutination and haemolysis scores between the first and the second sampling period. Groups are shown in the following order: healthy, activated, and sick.

Haemolysis and haemagglutination scores did not differ among groups or between sampling periods (Fig. 3 and Tables 1 and 2). The interaction between group and sampling period was not significant for both markers (Table 2). Males and females did not differ for both scores in the first (*F* < 0.02, *P* > 0.89) and in the second sampling period (*F* < 0.36, *P* > 0.55).

### Telomere length

**Figure 4:**
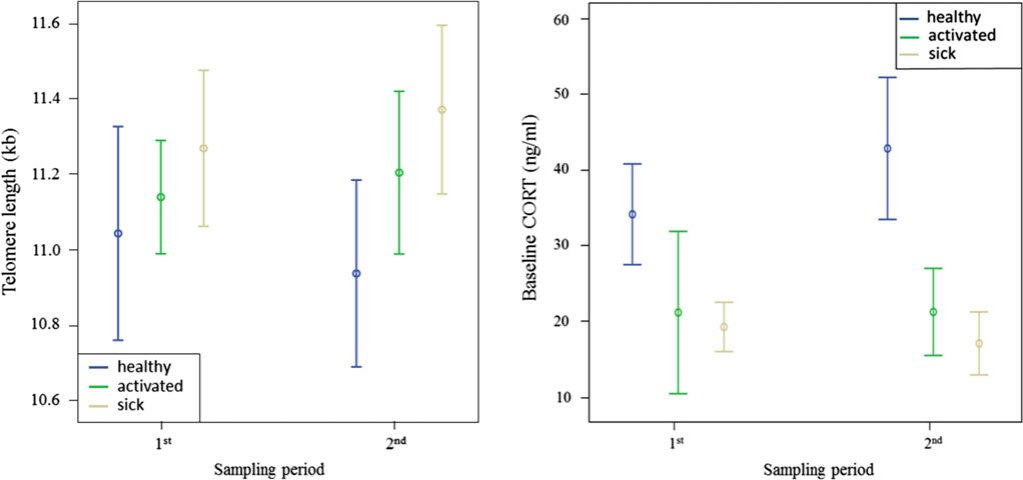
Mean values ± SEM of the telomere length and baseline corticosterone concentrations between the first and the second sampling period. Groups are shown in the following order: healthy, activated, and sick.

Telomere length did not differ among groups or between periods, and the interaction between group and sampling period was not significant (Fig. 4 and Tables 1 and 2). Furthermore, telomere length did not differ between males and females both in the first (*F* < 0.01, *P* = 0.93) and in the second sampling period (*F* = 1.74, *P* = 0.19). However, in the second sampling period, healthy nestlings had shorter telomeres than activated and sick nestlings grouped together (*t* = 2.67, *P* = 0.011).

### Plasma corticosterone

The plasma concentration of CORT did not differ between sampling periods, and the interaction between group and sampling period was not significant (Fig. 4 and Tables 1 and 2). Plasma CORT differed significantly among groups (Fig. 4 and Tables 1 and 2), with the sick group having less CORT than the healthy group (*t* = 2.51, *P* = 0.02; Fig. 4 and Table 1), whereas the activated group had similar CORT to that of the healthy group (*t* = 1.70, *P* = 0.10; Table 1) and of the sick group (*t* = 0.30, *P* = 0.77; Table 1). Corticosterone concentration did not differ between males and females both in the first (*F* = 0.90 *P* = 0.35) and in the second sampling period (*F* = 0.21, *P* = 0.65). Individuals with clinical signs had lower baseline CORT than individuals without clinical signs in the second sampling period.

### Markers as a tool to predict the occurrence of clinical signs and short-term survival

**Figure 5:**
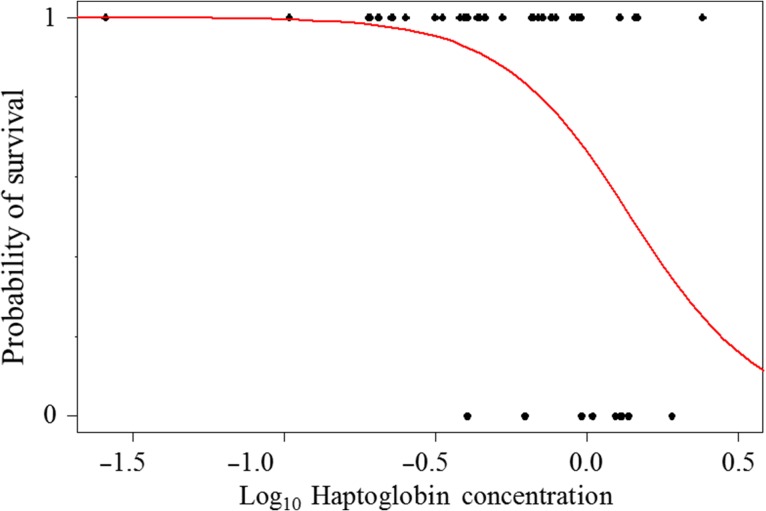
Survival probability of nestling frigatebirds (survival = 1 and non-survival = 0) in relationship to the concentrations of haptoglobin expressed as milligrams per millilitre.

Haptoglobin did not predict the occurrence of clinical signs (*Z* = 1.15, *P* = 0.25) or the short-term survival probabilities of sick nestlings (*Z* = −1.19, *P* = 0.23), but a higher concentration of haptoglobin meant lower survival perspectives for all nestlings regardless of their health status (*Z* = −2.6, *P* = 0.01; Fig. 5). Haemolysis and haemagglutination scores did not predict the occurrence of clinical signs (*Z* < 1.21, *P* > 0.23) or the short-term survival probabilities (sick group only, *Z* < 0.14, *P* > 0.89; and all groups, *Z* < 0.47, *P* > 0.64). Nitric oxide, corticosterone and telomere length did not predict the occurrence of clinical signs (*Z* < 0.80, *P* > 0.11) or the survival probabilities (sick group only, *Z* < −0.34, *P* > 0.63; and all groups, *Z* < 0.27, *P* > 0.65).

## Discussion

Our results showed that the plasma concentration of haptoglobin was greatly increased during a disease probably caused by herpesvirus infection and might predict the short-term probability of survival of frigatebird nestlings. Thus, haptoglobin might be a tool for assessment of the impact of herpesvirus on frigatebird nestlings. Our results also showed that individuals with clinical signs were not immunosuppressed, because the levels of complement and natural antibodies were similar among tested groups. Furthermore, contrary to our expectation, the basal plasma concentration of CORT did not increase in the ‘activated’ group and, generally, sick individuals had lower CORT than healthy nestlings. Finally, telomere length and nitric oxide did not seem to be affected by the viral activity, nor did they prove useful as markers to predict both the occurrence of visible clinical signs and short-term survival during this disease.

Previous studies have shown that haptoglobin can increase or decrease depending on the type of infection. For instance, an increased plasma concentration of haptoglobin was found in chickens infected by *Escherichia coli* and *Eimeria tenella* (Georgieva *et al*., 2010) or *Salmonella enterica* (Garcia *et al*., 2013), in domestic canaries exposed to *Plasmodium relictum* (Cellier-Holzem *et al*., 2010) or in wandering albatrosses (*Diomedea exulans*) with a higher density of *Ixodes uriae* on the head plumage (Costantini *et al*., 2015). Likewise, broiler chickens infected by bronchitis virus showed significantly higher haptoglobin than healthy individuals (Asasi *et al*., 2013), whereas Japanese quails (*Coturnix japonica*) infected by aspergillosis had a lower haptoglobin concentration than control birds (Goetting *et al*., 2013). However, irrespective of the direction of the change, there is a general consensus that haptoglobin concentrations change significantly in response to an inflammatory stimulus such as infection, injury or malignancy (Quaye, 2008). For those reasons, haptoglobin has recently been proposed as a predictor of both clinical signs and survival in humans and birds (Goetting *et al*., 2013; Sun *et al*., 2016). In our study, short-term survival probabilities of nestlings decreased drastically when the plasma concentration of haptoglobin exceeded 0.32 mg/ml (corresponding to −0.5 on the *x*-axis of Fig. 5). Thus, a quantification of haptoglobin in plasma could be a valuable tool to predict short-term survival perspectives of frigatebird nestlings facing a herpesvirus disease and, possibly, in other wild animals. Although most of the individuals with clinical signs are likely to die within a few weeks (personal observations, Pineau K.), the identification of a marker associated with survival probabilities might indicate which systems that regulate the physiological homeostasis are disrupted by the disease. In doing so, it is, for example, possible to define a more specific pharmacological treatment of the disease. Finally, the general increase in the haptoglobin concentrations that occurred from the first to the second sampling period is probably attributable to an inflammatory status resulting from the infection, which results in the increased synthesis of haptoglobin (Quaye, 2008). Indeed, individuals with clinical signs, and especially individuals from the activated group, were probably undergoing an aggressive stage of the disease during this period. It has been shown that cells facing an herpesvirus outbreak increase the expression of interleukins (Kuhnle *et al*., 1996; An *et al*., 2002), which in turn stimulate the expression of haptoglobin (Quaye, 2008).

Haptoglobin is also able to prevent nitric oxide scavenging from haemoglobin and to preserve nitric oxide signalling (Schaer *et al*., 2016). Nitric oxide is a small molecule that is highly reactive and diffusible, synthesized by oxidation of l-arginine by the inducible nitric oxide synthase or type 2 nitric oxide synthase (Bogdan *et al*., 2000). Given that the expression of these enzymes is remarkably increased when induced by inflammatory cytokines, microorganisms and reactive oxygen species (Arzumanian *et al*., 2003), the level of nitric oxide has been used in ecological studies as a measurement of innate immunity (Bourgeon *et al*., 2007) and a condition index during pathogenesis (Lillehoj and Li, 2004). Bourgeon *et al*. (2007) found an increase in the nitric oxide concentrations from 106 to 179 µmol/l in the common eider (*Somateria mollissima*) after lipopolysaccharide injection (Bourgeon *et al*., 2007), and a more recent study has found a significant increase in serum nitric oxide concentrations following virus infection in chickens and ducks (Burggraaf *et al*., 2011). However, although a previous review has highlighted a major role of nitric oxide during viral infections (Akaike and Maeda, 2000), in our study individual nitric oxide concentrations did not differ significantly between healthy and sick nestlings. For diagnostic purposes, nitric oxide also showed lower sensitivity and specificity than haptoglobin. Moreover, the combination of haptoglobin and nitric oxide, despite being efficient in identifying 100% of individuals from the activated group, lacked specificity. Possibly, for future studies, the combination of several markers could help to achieve a good balance between specificity and sensitivity, providing an efficient diagnostic tool for viral diseases.

In addition to haptoglobin and nitric oxide concentrations, we tested the capacity of frigatebird blood to agglutinate and lyse exogenous blood, which is a measurement of immunocompetence (Matson *et al*., 2005). Our study clearly showed that there were no significant differences among groups for both tests. This is surprising because viral infection is well known to stimulate the immune system in humans and laboratory animals (Vollstedt *et al*., 2004; Chew *et al*., 2009). Thus, we would have expected an increase in the immune response in the activated group in order to counteract the virus spread, or at least a difference in the immune markers between the healthy and the sick birds. The reason for an absence of up-regulation of the immune system might lie in the immunosuppressive action of glucocorticoids (Coutinho and Chapman, 2011) that are secreted in the event of infection in order to avoid both overstimulation of the immune defenses and immunopathology (Besedovsky and del Rey, 1996). However, our results did not support this explanation because plasma concentrations of CORT were not higher in sick than in healthy nestlings. On the contrary, CORT was higher in healthy than in sick nestlings, and this difference was more pronounced in the second sampling period. Here, we propose two explanations for the apparent lack of differences in the immune status between healthy and sick nestlings. First, it is possible that the immune traits we measured in apparently healthy nestlings might not reflect the true and basal immunocompetence of nestlings. In another study, we have shown how some healthy nestlings were positive for circulating herpes viral copies (Sebastiano *et al*., unpublished observations). Thus, it could be that any differences in immune traits between healthy and sick birds (including nestlings with the presence of herpes viral copies) have been masked because immune activity might have been up-regulated in some healthy nestlings. The information on the presence of circulating copies of herpesvirus, however, was obtained from a pilot study carried out to develop a new technique to assess the presence of the virus in individuals without visible clinical signs of a herpesvirus outbreak. Although these preliminary results confirm the presence of viral copies also in nestlings without clinical signs, the methodology is not yet well developed; therefore, some caution is still needed in drawing definitive conclusions. Second, the immune traits we have measured in our study might not have been responsive to this specific infection. Given the several mechanisms involved in the immune response, the simultaneous use of multiple immune metrics might help to cover the complexity of the immune system (Legagneux *et al*., 2014). Although the levels of complement and natural antibodies are amongst the most used in avian immunology, it would be interesting in future work to include additional markers, such as heterophile-to-lymphocyte ratio, avian immunoglobulin and basophils (Legagneux *et al*., 2014).

Regarding CORT, it is possible that the low CORT concentration in sick nestlings compared with healthy nestlings reflects an attenuation of the stress response with chronic stress. Chronic stress generally produces a chronic activation of the hypothalamic–pituitary–adrenal axis, which in turn leads to increased baseline CORT concentrations. A previous study carried out on captive European starlings (*Sturnus vulgaris*) has, however, shown a decrease in basal and stress-induced CORT concentrations with the onset and progression of chronic stress (Rich and Romero, 2005). As we lack information on the acute stress response of frigatebirds, we cannot explain whether the among group differences in CORT concentrations result from the attenuation of the stress response in sick nestlings or from an increase in basal CORT concentrations in healthy individuals.

Finally, our study showed that telomere length was not associated with the infection status. As stressful events can increase the rate at which telomeres shorten in both *in vivo* and *in vitro* studies (Epel *et al*., 2004; Boonekamp *et al*., 2014; Trusina, 2014), we expected that telomeres would be shorter in sick than in healthy nestlings. The lack of telomere loss might indicate that the time elapsed from the first to the second sampling period (14 days) was too short to cause a detectable shortening of telomere length. However, previous work found evidence of telomere shortening over both short (Meillère *et al*., 2015; Salmón *et al*., 2016) and long periods (Kotrschal *et al*., 2007; Boonekamp *et al*., 2014). If the disease were to cause telomere shortening, we should at least observe those individuals with visible clinical signs to have shorter telomeres than healthy nestlings. However, we also did not find evidence for a link between telomere length and infection status. A previous study has underlined a major role of chronic infection in telomere shortening, showing how great reed warblers (*Acrocephalus arundinaceus*) infected with chronic malaria have an accelerated telomere shortening in comparison with uninfected individuals (Asghar *et al*., 2015). Additionally, studies on humans have found herpesvirus to integrate into host telomeres and to cause telomere shortening and instability (Deng *et al*., 2014; Huang *et al*., 2014), in order to facilitate the release of viral genome from the chromosome (Huang *et al*., 2014). In our study, we found longer telomeres in individuals with visible clinical signs in the second sampling period. A possible explanation might lie with the fact that individuals with visible clinical signs of the disease were subjected to slower development in comparison to apparently healthy nestlings (field observation, Pineau K.). Indeed, previous studies have shown how telomeres might be shortened in individuals with faster development (Ringsby *et al*., 2015). Thus, a lower metabolic rate attributable to slower growth might have masked any effects of infection on telomere erosion. Further work will be needed to clarify whether the lack of association between infection status and telomere length we found in our study is dependent on growth rate.

## Conclusions

Our work provided strong evidence that the plasma concentration of haptoglobin is associated with clinical signs of the disease, and it is effective to predict short-term survival of frigatebird nestlings during a herpesvirus outbreak, supporting the use of haptoglobin as a diagnostic tool for viral diseases. Our study also showed that a single measurement of inflammatory and/or immune response might not be enough to assess the impact of viral infection on the health status of birds. Finally, our results suggested that there might be down-regulation of CORT during chronic viral infection. As with telomere length, although it did not appear to be linked to infection status, further study will be needed to assess whether this is attributable to a general slow growth rate and low metabolism of sick nestlings.
